# Synthesis of Novel Hyaluronic Acid Sulfonated Hydrogels Using Safe Reactants: A Chemical and Biological Characterization

**DOI:** 10.3390/gels8080480

**Published:** 2022-07-30

**Authors:** Elisa Sturabotti, Silvia Consalvi, Luca Tucciarone, Elisa Macrì, Valerio Di Lisio, Iolanda Francolini, Carmen Minichiello, Antonella Piozzi, Claudia Vuotto, Andrea Martinelli

**Affiliations:** 1 Department of Chemistry, Sapienza University of Rome, 00185 Rome, Italy; valerio.dilisio@dipc.org (V.D.L.); iolanda.francolini@uniroma1.it (I.F.); minichiello.1407329@studenti.uniroma1.it (C.M.); antonella.piozzi@uniroma1.it (A.P.); andrea.martinelli@uniroma1.it (A.M.); 2 IRCCS: Scientific Institute for Research, Hospitalization and Healthcare, Santa Lucia Foundation, 00179 Rome, Italy; silvia.consalvi@unicamillus.org (S.C.); lucatucciarone@gmail.com (L.T.); elisa.macri314@gmail.com (E.M.); c.vuotto@hsantalucia.it (C.V.); 3 UniCamillus-Saint Camillus International University of Health Sciences, 00131 Rome, Italy; 4 Donostia International Physics Center, Paseo Manuel de Lardizabal, 4, 20018 San Sebastian, Spain

**Keywords:** hyaluronic acid, crosslinking, sulfonation, mechanical properties, collagen, fibroblasts

## Abstract

Here, we present a one-pot procedure for the preparation of hyaluronic acid (HA) sulfonated hydrogels in aqueous alkaline medium. The HA hydrogels were crosslinked using 1,4-butanedioldiglycidyl ether (BDDE) alone, or together with N-bis(2-hydroxyethyl)-2-aminoethanesulfonic acid (Bes), as a safe sulfonating agent. Conditions for the simultaneous reaction of HA with BDDE and Bes were optimized and the resulting hydrogels were characterized under different reaction times (24, 72, and 96 h). The incorporation of sulfonic groups into the HA network was proven by elemental analysis and FTIR spectroscopy and its effect on water uptake was evaluated. Compared with the non-sulfonated sample, sulfonated gels showed improved mechanical properties, with their compressive modulus increased from 15 to 70 kPa, higher stability towards hyaluronidase, and better biocompatibility to 10T1/2 fibroblasts, especially after the absorption of collagen. As main advantages, the procedure described represents an easy and reproducible methodology for the fabrication of sulfonated hydrogels, which does not require toxic chemicals and/or solvents.

## 1. Introduction

Hyaluronic acid (HA) is a multipurpose material whose chemico-physical and biological key characteristics can be tuned deeply thanks to chemical modifications. From a chemical point of view, HA is a negatively charged linear polysaccharide composed of a disaccharide unit of D-glucuronic acid and N-Acetyl-D-glucosamine linked by β (1,4) and β (1,3) glycosidic bonds [1]. Nowadays HA, the only non-sulfated glycosaminoglycan present in the body is a biomaterial of particular importance in different fields, finding applications ranging from cosmetic to osteoarthritis disease treatments, to preparation of scaffold for tissue engineering, or micro- and nanogels for drug delivery [2,3,4]. For example, the overexpressed CD44 receptors for hyaluronic acid on tumor cells are widely exploited for targeted cancer therapy [5,6]. A number of modifications were suggested to confer to HA adequate viscosity or mechanical properties as well as to improve its biocompatibility and chemical or enzymatical stability. Crosslinking is a widely employed reaction to enhance hyaluronic acid viscosity for intra-articular viscosupplementation therapy, or for preparing insoluble hydrogel [7] and injectable formulations with in-situ gelation properties [8,9] for short- or long-term applications. Thanks to some key properties, including the presence of salient elements of native extracellular matrices (ECMs), mechanical properties similar to those of many soft tissues [10] and high permeability to nutrients and water-soluble metabolites, the HA hydrogels are suitable for the fabrications of scaffold for cell hosting, survival, and growth [11,12]. Anyway, some studies report that HA in the native form is substantially non-adhesive to cells, presumably because of it anionic state at physiological pH [13,14] and, thus, chemical interventions on HA are required to circumvent some of its drawbacks.

Hyaluronic acid has been adequately derivatized through reaction involving its carboxylic or hydroxyl group with molecules able to promote cell adhesion and proliferation [15], such as peptides [14] or dopamine [16]. Moreover, to create a more compatible environment for cells, HA hydrogels have been frequently combined with other bio-macromolecules, including collagen [17,18] and fibronectin, chitosan, and gelatin [13] through absorption promoted by electrostatic interaction, blending, or covalent conjugation.

As far as the enzymatic degradation of HA, it has been observed that it is decreased when the polysaccharide is crosslinked [19] or when sulfate (RO-SO_3_H) [20] or sulfonate groups (R-SO_3_H) [21] are incorporated into the hyaluronic acid backbone, because they can inhibit the activity of the enzyme by steric hindrance [22].

Due to their low pK_a_ ~ 1.7, sulfonate or sulfate groups are in anionic forms down to low pH whereas the carboxylic group is completely protonated. The negatively charged sulfonate or sulfate groups along the HA chain have been shown to have a direct effect on some biological processes (i.e., anti-inflammatory [23] and antiadhesive activity [24]) or to offer a stable site for the absorption of positively charged molecules or macromolecules by electrostatic interactions. This last property has been employed to favor the absorption of proteinaceous growth factors [25], of polycationic biomolecules, such as alginate, chitosan, and collagen [26,27], frequently used to increase cell adhesion, viability, and growth on scaffold surfaces.

Particularly, sulfated hyaluronic acid has been synthetized, converting its hydroxyl groups into RO-SO_3_H with a sulfur–pyridine complex in DMF [28], or by sulfonation with an oxidation/amidation process, drastically affecting the hyaluronate backbone [21]. Despite the high degree of functionalization, the few examples that deal with HA sulfonation and sulfation require the use of toxic reactants and solvents as well as laborious procedures for the water-organic solvents exchange and careful product purification.

In the present research, instead of direct link along the HA backbone, sulfonic groups were inserted into the hyaluronic acid network during the crosslinking reaction to provide the resulting hydrogel with anionic sites. This procedure was chosen for its simplicity, avoiding the organic solvents and toxic reactants. In general, the choice of safer synthetic procedures and chemicals, in fact, represents a challenging alternative to the traditional reaction methods that research is turning towards today. In addition to the operators’ safety, the purification phase of the product is simpler or avoidable. Furthermore, once the absence of material cytotoxicity is ascertained, the results of biological experiments are more reliable and the passage from research to product development is easier.

Specifically, crosslinking and sulfonation reactions were achieved in aqueous media by a simultaneous reaction of 1,4-butanedioldiglycidyl ether (BDDE), which is one of the most employed diepoxy crosslinker for HA biomedical uses [29], and N-bis (2-hydroxyethyl)-2-aminoethanesulfonic acid (Bes) as the sulfonating non-toxic agent. The idea at the base of this research is that Bes and HA hydroxy groups could be both involved in the reaction with the BDDE to form stable carbon–oxygen bonds, in basic condition. In this way, a conventional crosslinking procedure, such as that of BDDE, was exploited and improved to incorporate desired moiety into the polymeric network, leading to a material with adjustable features. Several experimental conditions were explored and properties of sulfonated samples were compared to those of non-sulfonated gels. The best formulations were characterized by water swelling and compression stress–-strain mechanical tests. Sulfonation was revealed by FTIR analysis while the morphology of dried hydrogels was studied by SEM microscopy. The resistance to enzymatic activity was evaluated following the weight loss of sample dwelled in hyaluronidase solution. Finally, the viability of 10T1/2 fibroblasts seeded on selected hydrogel samples, with or without collagen absorption, was analyzed by in vitro tests, while cellular morphology was observed using SEM. The one-pot crosslinking/sulfonation reaction was revealed to be a valid methodology for the synthesis of biocompatible HA-based materials.

## 2. Results and Discussion

**Figure 1:**
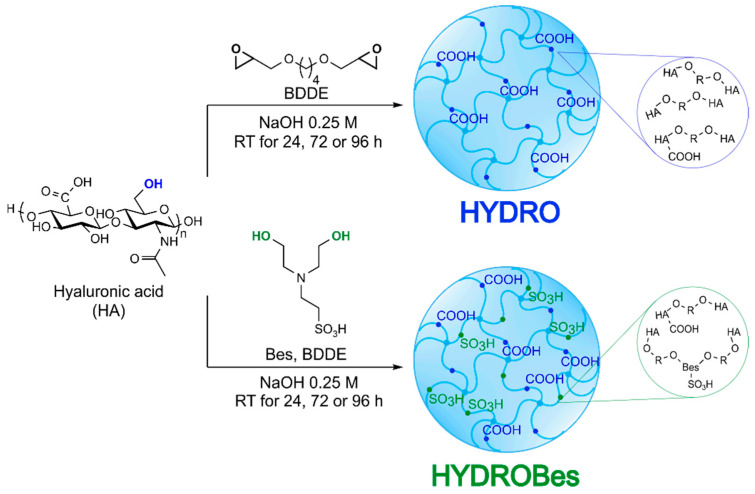
Scheme of synthetic conditions used for the preparation of hydrogel with BDDE (HYDRO) and BDDE/Bes (HYDRO-Bes), together with a schematic illustration of expected hydrogels (R = crosslinker). The HYDRO-Bes gels are enriched in -SO_3_H functionalities. Functional groups involved in the crosslinking/sulfonation reaction are evidenced.

Hyaluronic acid sulfonated networks were prepared through a one-pot procedure in water (Figure 1). For this aim, reaction between HA and crosslinker (BDDE) was carried out in the presence of a sulfonated agent (Bes) to simultaneously ring-open the di-epoxide by HA and Bes hydroxyl groups, in a strong alkaline medium (NaOH 0.25 M). The major advantage of this method is that the -SO_3_H moieties could be inserted into the hyaluronate network through the formation of stable ether bonds, exploiting the same condition required for the crosslinking (pH > 10) and, thus, achieving sulfonation of the polymeric matrix without the use of organic solvents or toxic reagents in a one-step synthesis. To the authors’ knowledge, the simultaneous ring-opening of a di-epoxide crosslinker has been proposed once for the chemical incorporation of collagen into hyaluronic acid matrices, thanks to the combined reaction of both biopolymers [30]. Crosslinking of HA is based on the covalent linkage of polysaccharide chains by BDDE through the formation of ether bonds. When Bes is added, the bifunctional sulfonating agent could react with epoxy rings of BDDE, already linked to HA, to form ether bonds (Figure 1). Therefore, the equivalents of Bes, BDDE, and HA should be properly adjusted to permit efficient crosslinking/sulfonation reactions, without an excessive consumption of the crosslinker by hyaluronic acid or Bes alone.

In addition to the crosslinking products displayed in Figure 1, it is worth specifying that the reaction of Bes or HA with BDDE could occur in one of the two available positions only and, accordingly, no contribution to the crosslinking is obtained. Moreover, any side product not linked to the HA backbone was easily removed during the dialysis.

In a preliminary study, various experiments were performed to obtain stable gels by varying the concentration of sodium hyaluronate solutions (5 wt%, 8 wt%, and 10 wt%) and Bes equivalents but maintaining the amount of BDDE constant. Crosslinking was carried out for 24, 72, or 96 h at room temperature in NaOH 0.25 M [31,32] (Table 1).

At the investigated highest HA concentration, the polysaccharide solution remained sufficiently fluid to ensure the homogeneous mixing of crosslinker. The observed low viscosity of sodium hyaluronate in 0.25 M NaOH (pH = 13.4) has been explained to be due to: (i) the competing mechanism of polymer coil expansion, due to the negative charge of –COO^-^ groups, and to the charge shielding caused by the high ionic strength of NaOH solution [33]; (ii) the reduced stiffness of the polymeric back-bone for the partial breakage of the H-bond network, favored by the −OH groups dissociation at pH > 12.5 [34]; (iii) HA molecular weight reduction as a result of pH-induced cleavage of glycosidic bonds [35]; or (iv) a combination of these events [36]. Unexpectedly, a drastic increase of viscosity was observed by the addition of Bes, although the solution underwent a low pH decrease (pH = 12.9). This effect, not investigated further in this research, presumably could be associated to a HA chain stiffening and coil expansion originated from the setup of intra- or inter-chain hydrogen bonds favored by the pH decrease and bridging Bes hydroxy groups. As shown later, this behavior plays a decisive role on the hydrogel properties.

In general, no gel formation or too soft and intractable hydrogels were obtained from 5 wt% or 8 wt% HA solutions (HA:Bes = 1:0.5), respectively. By increasing the sodium hyaluronate concentration up to 10 wt% and at the highest Bes molar ratios (HA:Bes = 1:1 and HA:Bes = 1:0.8), the homogenization of reaction mixture was difficult and, because of a presumable overconsumption of BDDE epoxy groups, the gelation did not occur. At HA:Bes = 1:0.5 molar ratio, a mechanically stable hydrogel was obtained, although further mixing time was necessary. For the sake of comparison, non-sulfonated hydrogels were also synthetized by using a 10 wt% HA solution and HA:BDDE = 1:1 molar ratio. No evident viscosity increase of sodium hyaluronate solution was observed by adding the crosslinker.

Sample names, reactant molar ratio, and crosslinking times (t_cross_) are summarized in Table 2.

The S/N molar ratio evaluated by elemental analysis of HYDRO-Bes samples crosslinked for 24, 72, and 96 h, was 0.26/3.28, 0.57/3.26, and 0.78/3.21, respectively. According to Equation (1), a degree of sulfonation of 0.03 (HYDRO-Bes-24), 0.09 (HYDRO-Bes-72), and 0.14 (HYDRO-Bes-96) was calculated. The data showed that by increasing the reaction time, the sulfur content of Bes in the gels increased to 0.14, which is less than the theoretical value of 0.5, expected from the molar ratio of the reagents used (Table 2).

**Figure 2:**
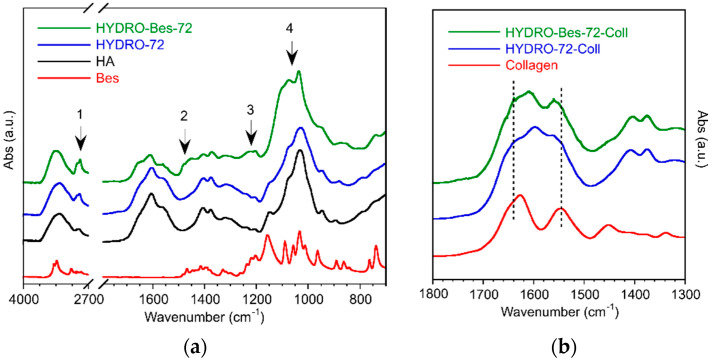
ATR-FTIR spectra of crosslinked gels: (**a**) HYDRO-72 and HYDRO-Bes-72 in the region between 4000 and 700 cm^−1^. The spectra are compared to those of pristine hyaluronic acid and Bes. (**b**) HYDRO-72-Coll, HYDRO-Bes-72-Coll, and collagen spectra in the 1800–1300 cm^−1^ region.

ATR-FTIR spectra were recorded on dry samples to investigate the hydrogels chemical structure after crosslinking and collagen absorption. No marked differences could be observed among the hydrogels obtained using different reaction period. As an example, the spectra of the sample crosslinked for 72 h (HYDRO-72 and HYDRO-Bes-72) together with those of HA and Bes are shown in Figure 2a. The same hydrogels after the collagen absorption are reported in Figure 2b.

The spectra of HA before and after reaction with BDDE are very similar since the bonds formed during the reaction are indistinguishable from those already present in the saccharide unit [37,38]. With respect to pristine HA, the networks showed a slight increase of aliphatic stretching at 2890 cm^−1^ (arrow 1 in Figure 2a), related to the increase of methylene groups from BDDE. In addition, the small decrease of OH stretching band at 3290 cm^−1^ and the absence of an ester peak at about 1750 cm^−1^ confirms that hydrogel formation at pH > 10 occurred solely via epoxide ring-opening by the hydroxyl functions and not by the carboxylate groups of HA [39]. As a result of Bes incorporation, a small but reproducible chance in HYDRO-Bes-72 spectra can be observed in the region between 1250 and 1200 cm^−1^, due to the S = O stretching band of -SO_3_H groups (arrow 3 in Figure 2a), also present in Bes spectra [40,41,42]. Moreover, the absorbance increases in the 1400–1500 cm^−1^ region can be assigned to the CH_2_ bending of BDDE and Bes (arrow 2 in Figure 2a). Finally, the band at 1090 cm^−1^ of the HYDRO-Bes-72 spectrum, due to C-N stretching, confirmed the presence of Bes (arrow 4 in Figure 2a).

The absorption of collagen was evaluated by the analysis of the spectral region between 1500 and 1800 cm^−1^, where the carbonyl stretching of HA carboxylate and of protein amide resonate. The spectra of HYDRO-72-Coll and HYDRO-Bes-72-Coll are reported in Figure 2b, together with that of pure collagen. The presence of the protein in both networks can be inferred by the increase of two shoulders at 1625 and 1545 cm^−1^ (Figure 2b: dashed lines) [43]. This spectral modification is more evident in the HYDRO-Bes-72-Coll sample because of the favored absorption of the protein by electrostatic interactions between the sulfonic group, in anionic form also at low pH, and the positively charged collagen chains.

**Figure 3:**
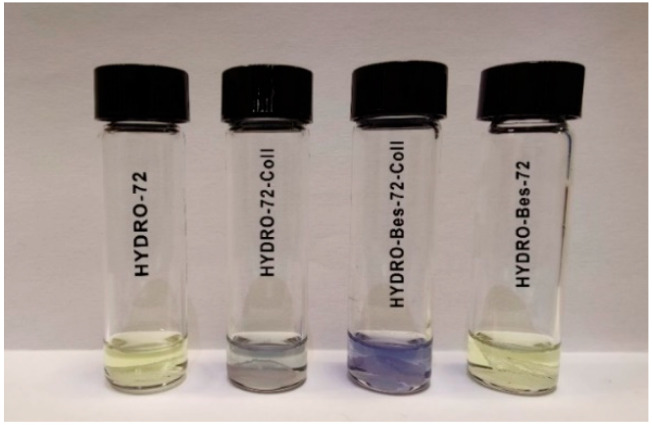
Images of ninhydrin test carried out on HYDRO-72, HYDRO-72-Coll, HYDRO-Bes-72-Coll, and HYDRO-Bes-72 dry samples.

A comparative ninhydrin test was carried out to evaluate the different collagen absorptions. Figure 3 shows the photographs of HYDRO-72-Coll and HYDRO-Bes-72-Coll reported together with those of the corresponding samples without collagen.

The images of Figure 3 show that no color change occurred in ninhydrin ethanol solution in contact with the samples without collagen. Differently, the presence of the protein brought about the formation of a blue-purple color, more intense in the vials with HYDRO-Bes-72-Coll. This let us infer a higher collagen amount absorbed into the sulfonate gel.

**Figure 4:**
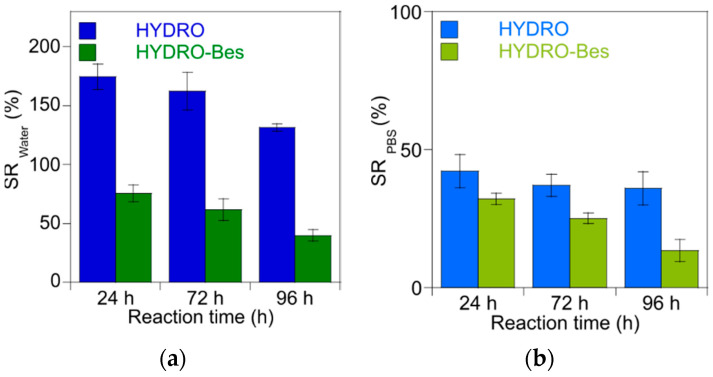
Swelling ratio (SR) of hydrogels measured at 37 °C after 24, 72, or 96 h of crosslinking: (**a**) SR in distilled water (**b**) in PBS.

The capability of non-sulfonated and sulfonated gels to absorb aqueous media was studied by soaking the dried sample in distilled water or PBS (pH = 7.4) until the swelling equilibrium was reached. The swelling ratios (SR, Equation (1)), measured as function of crosslinking time, are reported in Figure 4.

All the hydrogels showed high water uptake, increasing their dried weight about 50–180-fold when swollen in distilled water (Figure 4a). As expected, the water uptake in PBS was lower and the hydrogels reached a maximum weight 50 times higher than that of dry samples (Figure 4b), similarly to results reported by Baek et al. [44] for the HA-BDDE hydrogel. In general, by increasing the reaction time, the water swelling decreases, sign of an increased crosslink density [45]. SR for HYDRO 24, 72, and 96 h decreased from 175% to 130% while HYDRO-Bes samples displayed swelling values that ranged from 75%, 60%, to 40% within the 96 h. This effect nearly vanished in the swelling experiment in PBS, because the ion strength of the buffer implicates the contraction of the network [46]. The water swelling behavior is a complex phenomenon that depends deeply upon the crosslinking degree which, in turn, is affected by polymer network composition and crosslinking conditions. This aspect is particularly evident if the SR of non-sulfonated and sulfonated gels is compared. For HYDRO samples, the SD in water and, to a lesser extent, in PBS result as always higher than those of HYDRO-Bes, even if an opposite trend could be expected taking into account the increase of hydrophilicity provided by the -SO_3_^−^ groups [47]. Moreover, the possible formation of larger network meshes due to the presence of Bes molecule between the crosslinker and the HA chains can be expected (Figure 1). A possible interpretation of SR results could be given considering the viscosity increase observed after the addition of Bes to the HA-concentrated solution. In fact, it can be hypothesized that upon the addition of the sulfonated additive, a more expanded HA structure is formed, which possibly favors physical entanglement increase. Then, inter-chain crosslinks are more probable than ineffective intra-chain crosslinks [48]. It results in an enhancement of efficiency of BDDE and, consequently, in the formation of less swellable hydrogels [49].

**Figure 5:**
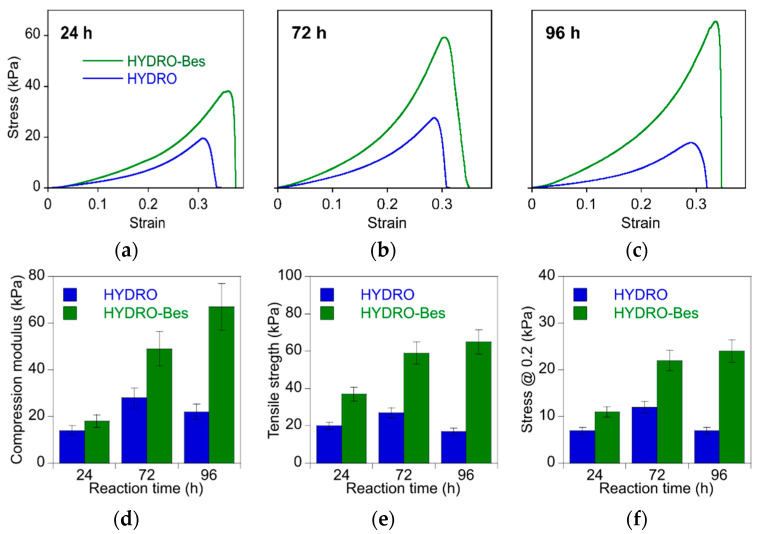
Mechanical characterization: (**a**–**c**) compression stress–strain curves for HYDRO and HYDRO-Bes at 24 h, 72 h, and 96 h of crosslinking, (**d**) compression modulus, (**e**) tensile strength, and (**f**) stress at 0.2 strain of HYDRO and HYDRO-Bes crosslinked for different times.

Differences in hydrogels’ structures were further evaluated testing their mechanical properties by stress–strain experiments in compression mode. The resulting curves and mechanical data of representative gels, crosslinked employing different reaction times, are displayed in Figure 5a–f, respectively.

For all the gels, the stress increased without inflection up to about 0.3–0.35 strain, after which the structure break-down occurred. The comparison of the stress–strain curves evidenced that the HYDRO-Bes samples are consistently tougher than HYDRO samples. To further investigate this aspect, the mean values (*n* = 3 ± SD) of the compression modulus, calculated as the mean slope of the straight line interpolating the data between 0 and 0.2 strain, of the tensile strength and of the stress at 0.2 strain, were calculated and the results are reported in Figure 5d–f.

The HYDRO-Bes gels resulted to have a systematically higher toughness than that of HYDRO samples. For the sulfonated material, the compression modulus increased from 60 to 105 kPa at the maximum time of crosslinking. Additionally, tensile strength and stress at fixed deformation increased progressively with time. In contrast, no marked differences were displayed by the HYDRO-24, HYDRO-72, and HYDRO-96, and, for example, the elastic modulus did not exceed 30 kPa. Since the mechanical features and the swelling behavior are both strictly related to the hydrogel structure [45], correlations between the stress–strain results and SR could be made. In fact, it can be observed that all the samples showed a stiffness increase with the swelling decrease. In particular, a good correlation exists among the HYDRO-Bes samples. Then, it could be assumed that the use of Bes as sulfonate additive favors the synthesis of gels with enhanced mechanical stability and characterized by Young’s moduli comparable to those of scaffolds for soft tissues regeneration [50,51].

**Figure 6:**
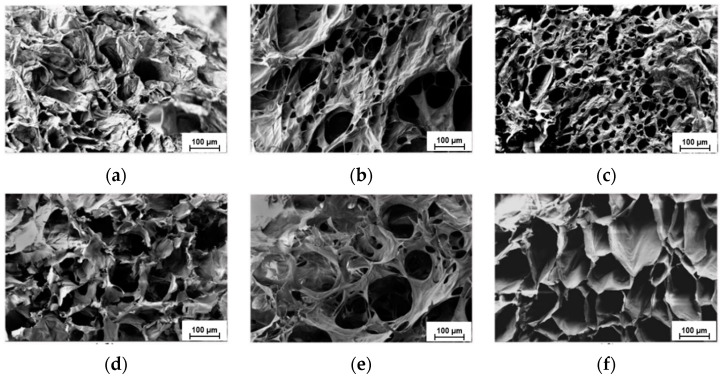
Scanning Electron Microscopy (SEM) micrographs of HYDRO (**a**–**c**) and HYDRO-Bes (**d**–**f**) crosslinked for 24 h (**a**,**d**), 72 h (**b**,**e**), and 96 h (**c**,**f**).

An adequate procedure for the preparation of hydrogels should be able to form porous and interconnected structures, ensuring the diffusion of nutrients and metabolic products, allowing them to host cells, and promoting their proliferation. Therefore, the morphology of non-sulfonated and sulfonated hydrogels was investigated by scanning electron microscopy (SEM). The images of freeze-dried gels’ cross-sections, prepared at 24, 72, and 96 h of crosslinking, are displayed in Figure 6.

The micrographs show that HYDRO samples display a great dimension heterogeneity of pores and low pore interconnection, mainly at the shorter crosslinking time. The structures appeared collapsed in some points, probably as result of freeze-drying. On the other hand, HYDRO-Bes-24 and HYDRO-Bes-72 samples were characterized by a more open and regular structure with a lower dispersion of pore dimension. The shape of pores changed with crosslinking time. Specifically, HYDRO-Bes-96 showed oriented tubular pores with compact and continuous walls. However, the different morphologies observed between HYDRO and HYDRO-Bes hydrogels could be in part related to the sample rigidity that, when increased, prevents the structure crumple upon drying. The hydrogel obtained at 72 h of crosslinking was considered to have, beside good mechanical stability, the most suitable structure for tissue engineering considering that, generally, pores between 70 and 150 μm are necessary for the regeneration of soft tissues [52,53,54]. Then, this sample, together with its non-sulfonated respective was used for the following enzymatic degradation and biological experiments.

**Figure 7:**
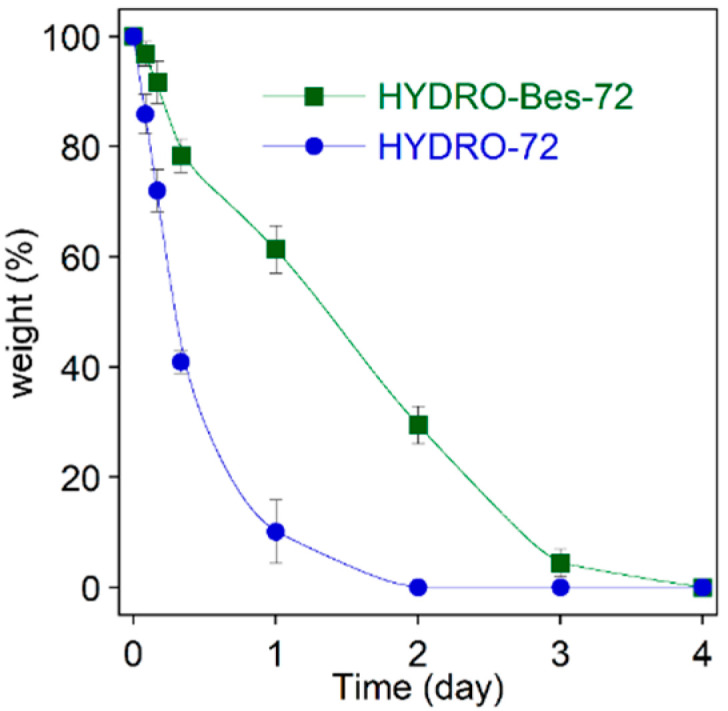
HYDRO-72 and HYDRO-Bes-72 degradation by hyaluronidase in PBS at 37 °C.

The enzymatic degradation of HYDRO-72 and HYDRO-Bes-72 was tested in the presence of hyaluronidase (100 U/mL) at 37 °C. The residual mass (*n* = 3 ± SD) of both samples as a function of time is compared in Figure 7.

As expected, the hydrogels underwent a weight decrease, although the weight loss rate of HYDRO-72 resulted to be higher than that observed for HYDRO-Bes-72. During the first 4 h, the non-sulfonated sample lost about 25% of the initial weight, while HYDRO-Bes-72 lost the 15%. After 1 day, HYDRO-72 almost solubilized whereas the sulfated hydrogel degraded completely within 3 days. The enzymatic degradation of hydrogels can be associated with many factors, including chemical composition, morphology, and crosslinking density [55]. From the comparison of degradation profiles with the structural properties, it can be inferred that the faster degradation process taking place in HYDRO-72 is related to its lower crosslink density, which also involves the higher water uptake values and the lower mechanical stability. On the other hand, HYDRO-Bes, which has a tighter and stiffer structure, can resist the diffusion and lysis of the enzyme better, offering a more stable matrix towards external agents. The increases in steric hindrance determined by sulfonation is often considered to inhibit hyaluronidase digestion [22]. Therefore, besides the structural aspects, the resistance towards the enzyme could also be related to the presence of sulfonic groups, although they are not directly linked to the HA backbone.

**Figure 8:**
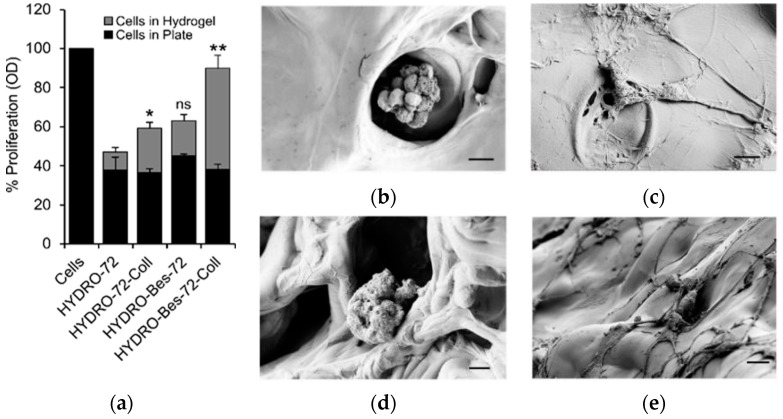
In vitro biological experiments: (**a**) cell viability measured by MTT assay on 10T1/2 fibroblasts cultured in test well bottom (control) and in presence of HYDRO-72, HYDRO-72-Coll, HYDRO-Bes-72, and HYDRO-Bes-72-Coll for 72 h. SEM images of fibroblasts cultured on HYDRO-72 (**b**), HYDRO-72-Coll (**c**), HYDRO-Bes-72 (**d**), HYDRO-Bes-72-Coll (**e**) for 72 h. Significant difference is showed by statistical analysis when * *p* ≤ 0.05 and ** *p* ≤ 0.01. ns = not significant.

In light of the abovementioned results, the cytocompatibility of the samples crosslinked for 72 h was tested with 10T1/2 fibroblasts by direct contact method [56]. Cells were seeded onto HYDRO-72, HYDRO-72-Coll, HYRDO-Bes-72, and HYDRO-Bes-72-Coll and MTT assay was used to evaluate cell proliferation after 72 h experiments (Figure 8a).

Cells adapted to the hydrogels evidenced the non-toxic character of the matrices. After the seeding, a fraction of cells did not adhere onto the hydrogel but to the well bottom. For this reason, the MTT assay was performed both on cells’ growth inside the hydrogels (Figure 8a, grey bars) as well as on polystyrene plate bottom (Figure 8a, black bars). Cells in the test wells without hydrogel were used as control. The comparison of the data obtained from the different samples and between the two sampling sites can help to distinguish the role of hydrogel compositions on cell proliferation. In general, it can be observed that the overall proliferation depends largely on the cells in the hydrogel since the contribution of those in the well bottom is almost constant (~40%, *p* > 0.05). Moreover, Figure 8a shows that the number of engrafted cells increased when collagen was absorbed onto the hydrogels. Worthy of note, therefore, is the significant increase in cell proliferation in HYDRO-Bes-72-Coll thanks to the higher absorption of collagen, favored by the electrostatic interaction of the protein with the sulfonate anion. In addition to viability, the morphological evolution of the fibroblasts seeded in the gels is another important indication for assessing the aptitude of the scaffold to accommodate cells. In Figure 8, SEM images of hydrogels samples after 72 h of culture are reported. Both the samples without collagen showed that the cells maintained their spherical shape and tended to aggregate (Figure 8b,d), signs of an unfavorable interaction with the substrate. This behavior could be due to the anionic nature of HA and sulfonated HA which has been reported to hinder the cell attachment [12,44]. The fibroblasts on the hydrogels with collagen, on the other hand, underwent morphological changes (Figure 8c,e). The cells, in fact, protruded filopodia and evolved towards a flat and elongated shape to favor good contact among them and with substrate. The morphological transformation is more evident for cells cultured on HYDRO-Bes-72-Coll due to a greater surface coverage of the absorbed protein and, possibly, to the greater rigidity of the gel, which ensures a more stable substrate for fibroblast adhesion (Figure 8e).

## 3. Conclusions

In the present study, a one-pot synthesis of sulfonated networks from soluble hyaluronic acid was presented to provide HA-based hydrogels with anionic sites to favor the absorption of collagen and, then, to ameliorate cell adhesion and growth.

The set-up for the reaction in water was described using BDDE as crosslinking agent. The grafting of -SO_3_H groups was accomplished by the insertion of Bes in the network structure, in parallel to crosslinking. Differently from other methods used to insert sulfate or sulfonic group directly along HA backbone, the simultaneous sulfonation and gel formation was achieved in water by using non-toxic reactants. This allowed the rapid and facile purification of hydrogels by dialysis and avoided complex organic solvent/water exchange procedures. Compared to the hydrogels crosslinked with BDDE alone, the observed advantages of including of Bes in the HA network are:

- the formation of a more regular porous structure with interconnected pores;
- a decrease of the equilibrium water uptake and, therefore, an increase of gel mechanical stability;
- a higher hydrogel stability towards enzymatic degradation;
- a greater collagen absorption by electrostatic interaction of the protein with the sulfonic groups, in anionic form even at low pH;
- a resulting increase of viability of fibroblasts cultured for 72 h into the hydrogel with collagen;
- a more stable attachment of fibroblasts on the gel surface, as evidenced by cell morphological changes and formation of filopodia.

In summary, the facile and safe synthesis as well as adequate chemical, mechanical and biocompatibility properties make HYDRO-Bes gels good candidates for tissue engineering and wound-dressing fields as well as in all the applications in which negative charges in the network are needed. They can be exploited, for example, to prepare antiadhesive surfaces to prevent postsurgical adhesion, or as nano- or microgel carriers for the loading and release of cationic drugs.

## 4. Materials and Methods

### 4.1. Materials

Sodium hyaluronate (HA) with a molecular weight of 1200–1500 kDa was purchased from Flower tales (Milan, Italy). Before any use, HA was dialyzed for 48 h against double-distilled water in a membrane with a 14 kDa cut-off. N,N-bis(2-hydroxyethyl)-2-aminoethanesulfonic acid (Bes), 1,4-butanedioldiglycidyl ether (BDDE), sodium hydroxide, saline phosphate-buffered (PBS) and hyaluronidase (type IV-S from bovine testes, 750–3000 U/mg) were obtained from Sigma-Aldrich (Milan, Italy). Collagen (Atelocollagen sponge) was purchased from Cosmo Bio Co. (Tokyo, Japan). All reactants were used without any further purification.

### 4.2. Hydrogel Preparation

Water-insoluble hydrogels were obtained after preliminary experiments carried by varying HA and Bes concentration. In the selected reaction conditions, to a 10% (wt/v) sodium hyaluronate in 0.25 M NaOH solution, BDDE or BDDE, and Bes were added in 1:1 (HA:BDDE) [3,32,57,58,59] and 1:1:0.5 (HA:BDDE:Bes) molar ratios to obtain hyaluronate (HYDRO) and hyaluronate-sulfonated hydrogels (HYDRO-Bes), respectively. The mixtures were stirred and the homogeneous solutions were poured into Teflon cylindrical molds (internal diameter 0.6 cm). Then, samples were sonicated for 30 min at 40 °C to remove possible air bubbles. After 24, 72, or 96 h at room temperature, the hydrogels were soaked in a large amount of distilled water for 3 days to remove unreacted molecules. Finally, the hydrogels were quenched in liquid nitrogen, freeze-dried, and stored at 4 °C until further use. The reaction time was chosen according to the indication of Baek et al. (2018) [44], who found that the crosslinking at room temperature prevails in the first 4 days of reaction, before the HA degradation process takes over. According to its properties, the 72 h type hydrogels were selected for the biological tests. For this aim, freeze-dried sponges were soaked in a 0.1 wt% collagen solution at pH 4 for 2 h, washed three times with 10 mL of distilled water, and freeze-dried. Sample names, reactant molar ratio, and crosslinking times (t_cross_) are summarized in Table 2.

### 4.3. Degree of Sulfonation Determination

The degree of sulfonation was evaluated from the S/N molar ratio of HYDRO-Bes samples obtained from elemental analysis (EA 1110 CHNS-O). Taking into account that each HA repeating unit bears one nitrogen atom and that S/N = 1 for Bes, the sulfonation degree can be evaluated by the relation:

(1) Degree of sulfonation=molS(molN−molS)

where (N-S) is the nitrogen of HA repeating unit in HYDRO-Bes.

### 4.4. Infrared Spectroscopy (FTIR-ATR)

FTIR spectroscopy was used to characterize crosslinked and sulfonated hydrogels with or without collagen absorption. The spectra of freeze-dried samples were acquired in attenuated total reflection mode (ATR) using a Nicolet 6700 (Thermo Fisher Scientific, Waltham, MA, USA) equipped with a Golden Gate single reflection diamond ATR accessory (Specac, Orpington, UK). All spectra were recorded with 200 scans/spectrum at 4 cm^−1^ resolution.

### 4.5. Ninhydrin Test

4 mg of dry HYDRO-72 and HYDRO-Bes-72, before and after collagen deposition, were immersed in 1.5 mL solution of ninhydrin in ethanol (10 mg/mL). Ninhydrin was allowed to react at 90 °C for 15 min and then samples were cooled at room temperature. Photographs of samples were captured to qualitatively estimate the amount of absorbed collagen on the sulfonated and non-sulfonated hydrogels.

### 4.6. Water Swelling Determination

Water uptake properties of hydrogels were studied by gravimetric measurements following the equilibrium swelling ratio (SR) in distilled water and PBS 0.01 M. Freeze-dried specimens were weighed (dry weight, *W_d_*) and soaked in 50 mL of the specific medium at 37 °C for 24 h, time enough to reach the equilibrium swollen state. Then, the samples were removed from the solutions, lightly patted with absorbent paper to eliminate the excess water, and weighed again (swollen weight, *W_s_*). The swelling ratios (SR) in the two media were calculated as follows:

(2) $$\mathrm{SR}=\frac{W_{s}}{W_{d}}\times 100$$

Swelling measurements were carried out in triplicate and final values were reported as means ± standard deviations.

### 4.7. Mechanical Measurements

Uniaxial compression tests on swollen hydrogels were carried out at RT using an INSTRON 4502 instrument (Instron Inc., Norwoon, MA, USA). Cylindrical samples, (base diameter 6 mm, height 6 mm) were placed between the two flat plates and compressed at 5 mm/min^−1^. The applied force was recorded by a 10 N load cell. Compression moduli (CM) and stresses at a fixed deformation (*σ_ε_*) were determined from stress–strain curves obtained by plotting nominal stress *σ_n_* as function of the deformation ε. In particular, CM was calculated from the slope of the linear zone of the stress–strain curve in the range 0 < *ε* < 0.2 while the stress at constant deformation was evaluated at *ε* = 0.2. The results are reported as average values ± standard deviations on at least three experiments conducted on different specimens.

### 4.8. SEM Morphological Analysis

The morphology of hydrogels’ cross-section was studied by field emission scanning electron microscopy (FESEM, AURIGA Carl Zeiss AG, Oberkochen, Germany). Lyophilized samples were sputtered with gold before the analysis.

### 4.9. In Vitro Degradation

Enzymatic degradation of non-sulfonated and sulfonated hydrogels was evaluated using hyaluronidase (HYAL type IV-S). In particular, freshly prepared hydrogels were soaked in PBS for 24 h at 37 °C and, then, incubated at 37 °C in the hyaluronidase solution (100 U/mL in PBS). At specific time intervals, the samples were removed from the medium, wiped, and weighed (weight at specific time, *W_t_*). The residual mass percentage was determined as follows:

(3) $$\mathrm{Residual}\;\mathrm{mass}\;(\%)=\frac{W_{t}}{W_{i}}\times 100$$

where *W_i_* is the initial weight of sample swollen in PBS. The results are reported as average values ± standard deviations on at least three experiments conducted on different specimens.

### 4.10. Cell Culture

Mouse 10T1/2 fibroblasts were purchased from American Type Culture Collection (ATCC). Cells were cultured in a humidified atmosphere (5% CO2, 95% air) at 37 °C and propagated in Dulbecco’s Modified Eagle Medium (DMEM) (D4947, Sigma) supplemented with 10% FBS. Cells were cultured alone or in combination with hydrogels (200,000 cells seeded in 24-well plates with 1 mL of culture media). After 72 h, cells and hydrogels were harvested for MTT assay and SEM analysis.

### 4.11. MTT Assay

MTT (3-(4,5-dimethylthiazol-2-yl)-2,5-diphenyltetrazolium bromide) assay was performed to evaluate the proliferation of mouse 10T1/2 fibroblasts when seeded into hyaluronic acid hydrogel. MTT was carried out separately on cells engrafted into the hydrogels and on those adherents to the bottom of the culture plate. Cells seeded into a plate without the hydrogel were used as control. Briefly, adherent cells and hydrogels were incubated in 200 μL of serum-free media and 200 μL of MTT solution (5 mg mL^−1^ in PBS) at 37 °C for 3 h. After incubation, 600 μL of MTT solution (4 mM HCl, 0.1% NP40 in isopropanol) was added and plates were wrapped in foil and shaken on an orbital shaker for 15 min. Optical density (OD) was read at 590 nm within 1 h and corrected by subtracting the culture medium background. Cell proliferation data were evaluated by the following equations:

(4) $$\mathrm{Proliferation}\;(\%)=\frac{{\mathrm{OD}}_{sample}}{{\mathrm{OD}}_{control}}\times 100$$

### 4.12. Cells Morphology (SEM)

Selected hydrogel samples (HYDRO-72, HYDRO-72-Coll, HYDRO-BES-72, and HYDRO-BES-72-Col) with fibroblasts cultured 72 h were fixed in 2.5% buffered glutaraldehyde solution at pH 7.3, for 3 h at room temperature, washed in the same buffer, dehydrated through a graded series of water-ethanol mixtures and finally with hexamethyl-disilazane (Fluka). SEM images of the gold sputtered samples were acquired by a Field Emission Scanning Electron Microscopy (AURIGA Carl Zeiss AG, Oberkochen, Germany).

### 4.13. Statistical Analysis

Data are presented as average ± SE (*n* = 3, biological replicates). These notations were used: ns = not significant; (*) indicates statistical analysis by Student’s t-test in the hydrogel comparison, * *p* ≤ 0.05, ** *p* ≤ 0.01.

## Figures and Tables

**Table 1:** Preliminary conditions used to optimize the crosslinking reactions.

HA (wt/v%)	HA:BDDE:Bes (Molar Ratio)	t_cross_ (h)	Gel Formation
5	1:1:0.5	24, 72, 96	No
8	1:1:0.5	24, 72, 96	Yes (soft gel)
10	1:1:0.5	24, 72, 96	Yes
10	1:1:0.8	24, 72, 96	No
10	1:1:1	24, 72, 96	No

**Table 2:** Hydrogel name, reactant molar ratio, and crosslinking time (t_cross_).

Sample	HA:BDDE:Bes * (mol)	t_cross_ (h)	Collagen
HYDRO-24	1:1:0	24	-
HYDRO-72	1:1:0	72	-
HYDRO-96	1:1:0	96	-
HYDRO-72-Coll	1:1:0	72	Yes
HYDRO-Bes-24	1:1:0.5	24	-
HYDRO-Bes-72	1:1:0.5	72	-
HYDRO-Bes-96	1:1:0.5	96	-
HYDRO-Bes-72-Coll	1:1:0.5	72	Yes

* The mole of HA refers to the polysaccharide repeating unit.

## Data Availability

Not applicable.
